# Physical/mechanical and antibacterial properties of orthodontic adhesives containing Sr-bioactive glass nanoparticles, calcium phosphate, and andrographolide

**DOI:** 10.1038/s41598-022-10654-6

**Published:** 2022-04-22

**Authors:** Wirinrat Chaichana, Kanlaya Insee, Supachai Chanachai, Sutiwa Benjakul, Visakha Aupaphong, Parichart Naruphontjirakul, Piyaphong Panpisut

**Affiliations:** 1 Division of Orthodontics, Faculty of Dentistry, Thammasat University, Pathum Thani, 12120 Thailand; 2 Division of Oral Biology, Faculty of Dentistry, Thammasat University, Pathum Thani, 12120 Thailand; 3 Biological Engineering Program, Faculty of Engineering, King Mongkut ’s University of Technology Thonburi, Bangkok, 10140 Thailand; 4 Division of Restorative Dentistry, Thammasat University, Pathum Thani, 12120 Thailand; 5 Thammasat University Research Unit in Dental and Bone Substitute Biomaterials, Thammasat University, Pathum Thani, 12120 Thailand

**Keywords:** Nanomedicine, Composite resin, Dental biomaterials, Dentistry, Orthodontics, Fixed appliances

## Abstract

White spot lesions around orthodontic brackets are the major complication during fixed orthodontic treatment. This study prepared orthodontic adhesives for promoting mineral precipitation and reducing bacterial growth. Adhesives with added calcium phosphate monohydrate/Sr-bioactive glass nanoparticles (Sr/CaP) and andrographolide were prepared. The physical/mechanical and antibacterial properties of the adhesives were tested. The additives reduced the monomer conversion of the materials (62 to 47%). The addition of Sr/CaP and andrographolide increased the water sorption (from 23 to 46 μg/mm^3^) and water solubility (from 0.2 to 5.9 μg/mm^3^) but reduced the biaxial flexural strength (from 193 to 119 MPa) of the adhesives. The enamel bond strengths of the experimental adhesives (19–34 MPa) were comparable to that of the commercial material (*p* > 0.05). The Sr/CaP fillers promoted Ca, Sr, and P ion release and the precipitation of calcium phosphate at the debonded interface. An increase in the Sr/CaP concentration enhanced the inhibition of *S. mutans* by 18%, while the effect of andrographolide was not detected. The abilities of the adhesives to promote ion release, calcium phosphate precipitation, and the growth inhibition of cariogenic bacteria were expected to reduce the occurrence of white spot lesions. The additives reduced the physical/mechanical properties of the materials, but the corresponding values were within the acceptable range.

## Introduction

The common complication during fixed orthodontic treatments is initial enamel caries or white spot lesions around the brackets^1^. These lesions occur due to the continuation of tooth demineralization and the dysbiotic dental biofilm^2^ caused by suboptimal oral hygiene during the treatment. If the carious lesions are left untreated, the lesions may progress and become deep and uncleanable cavities. This may subsequently lead to severe tooth infection/pain, complicating the orthodontic treatment. It was reported that the irregular surfaces of excess orthodontic adhesives promoted the accumulation of dental biofilms^3^. Hence, adhesives that offer remineralization and antibacterial actions may be needed to reduce the risk of dental caries. However, the currently available and commonly used resin composite orthodontic adhesives showed no anti-caries actions. Additionally, a suboptimal light-curing technique may increase the risk of releasing unreacted monomers^4^, which may promote the cariogenicity and microbial dysbiosis of dental biofilms^5^.

Various methods have been employed to enhance the remineralization and antibacterial actions of orthodontic adhesives. For example, glass ionomer cements were employed to facilitate fluoride release, enhancing caries resistance via the fluoridation of the tooth surface^6^. However, a clinical study reported that a significant reduction in tooth demineralization was not detected when using glass ionomer cement orthodontic adhesive^7^. Calcium phosphates can be used as reactive fillers to encourage the release of calcium and phosphate ions from orthodontic adhesives. These ions are essential for promoting suitable conditions for the precipitation of tooth minerals, such as hydroxyapatite^8,9^. Commercially available monocalcium phosphate monohydrate (MCPM) was incorporated into resin-based materials in previous studies^10,11^. The use of MCPM at high concentrations (10–20 wt%) substantially enhanced the precipitation of hydroxyapatite^10–12^, which was expected to promote remineralizing effects at the tooth-composite interface. However, the high solubility of MCPM (Ca/P ratio = 0.5) could lead to excessive water sorption, polymer plasticization, and significant reductions in the mechanical strengths of orthodontic adhesives^12^.

An alternative remineralizing agent to calcium phosphate compounds could be sol–gel bioactive glasses^13^. It was demonstrated that the incorporation of bioactive glass nanoparticles in orthodontic adhesive resulted in effective remineralizing actions^14^. Additionally, the use of spherical bioactive glass particles exhibited greater mineralizing effects compared to the use of irregular/granular shaped particles^15^. It was proposed that Sr^2+^ could potentially enhance apatite precipitation by increasing the number of nucleation clusters^16,17^. Additionally, it was demonstrated that bioactive glass doped with Sr exhibited superior antibacterial actions compared to non-Sr-doped bioactive glass^18^. The antibacterial actions of Sr^2+^ may involve the inhibition of the growth, cell wall synthesis, metabolism, and DNA replication of bacteria^18^.

The addition of antibacterial agents was expected to reduce biofilm colonization around orthodontic brackets. Studies have shown that the addition of chlorhexidine into orthodontic cements enhances antibacterial actions without affecting the bonding performance^19,20^. However, severe allergic reactions to chlorhexidine have remained a major concern^21^. Andrographolide (Andro) is a medicinal extract from *Andrographis paniculata* (Acanthaceae) that is traditionally used in herbal medicine. Andro is a bicyclic diterpenoid containing ɣ-lactone at both ends of the molecule. It exhibits various beneficial biological activities, such as anti-inflammatory, antiviral, antitumor, antioxidant, and antibacterial properties^22^. It was reported that Andro reduced biofilm colonization by interfering with the quorum-sensing system, thus inhibiting the formation of bacterial biofilms, the production of virulence factors, and bacterial coaggregation^23^. Additionally, a study demonstrated that Andro prevents the adherence of cariogenic bacteria (*S. mutans*) to hydroxyapatite beads by decreasing glucosyltransferase activity and eliminating the activity of glucan-binding lectin from strains^24^.

Currently, studies that investigate the potential use of low-toxicity and antibacterial herbal extracts such as Andro to enable antibacterial actions for orthodontic adhesives are limited. The aim of the current study was to prepare experimental orthodontic adhesives containing Sr-BGNPs/MCPM and Andro. The physical/mechanical properties of the materials and the inhibition of *S. mutans* by the materials were assessed. Additionally, the effects of increasing the concentrations of Sr-BGNPs/MCPM and Andro on the tested properties of the materials were analyzed. The hypothesis was that the increase of additives should not significantly affect the tested properties of the materials. All methods were carried out in accordance with relevant guidelines, protocols, and regulations.

## Materials and methods

### Preparation and characterization of Sr-bioactive glass nanoparticles (Sr-BGNPs)

Spherical Sr-bioactive glass nanoparticles (Sr-BGNPs) with a diameter of 200 nm were synthesized through a sol–gel process according to a method used in previous studies^25–27^. Silica nanoparticles (SiO_2_-NPs) were synthesized prior to cationic incorporation. Briefly, 0.32 M ammonium hydroxide, 6 M Milli-Q water, and 14 M ethanol (99.5%) were mixed in a 500 ml Erlenmeyer flask and stirred at 500 rpm for 10 min. Then, 0.28 M tetraethyl orthosilicate (TEOS) was gradually added to the prepared solution. The mixed solution was stirred for 10 h to complete the hydrolysis and polycondensation reactions. SiO_2_-NPs were collected and incorporated with 0.09 M calcium nitrate tetrahydrate (99%) and 0.27 M strontium nitrate (99%). The prepared particles were then calcined at 680 °C for 3 h at a heating rate of 3 °C/min. Particles were then cleaned with ethanol twice. The morphology and size of the particles were characterized using a scanning electron microscope. The composition of the particles was measured by using X-ray fluorescence (XRF, XUV773, Fischer Instrumentation, Worchestershire, UK) with X-ray generators in the range 8–20 kV operated in a vacuum. A diffractometer was used to identify the crystal diffraction pattern of the particles. The X-ray diffraction (XRD, Bruker, Massachusetts, USA) pattern was collected with a Bruker AXS automated powder diffractometer using Cu Kα radiation (1.540600 Å) at 40 kV and 40 mA. Data were collected in the 5–70° 2θ-range with a scan rate of 3°/min.

### Preparation of orthodontic adhesives

The experimental orthodontic adhesives were prepared using the protocol of a previous study^12^. Briefly, the liquid phase of the adhesives contained 70 wt% urethane dimethacrylate (Sigma–Aldrich, St. Louis, MO, USA), 26 wt% triethyleneglycol dimethacrylate (Sigma–Aldrich), 3 wt% 2-hydroxyethyl methacrylate (Sigma–Aldrich), and 1 wt% camphorquinone (Sigma–Aldrich). The powder phase contained silane-treated boroaluminosilicate glass (Esstech, Inc. Essington, PA, USA), Sr-bioactive glass nanoparticles (Sr-BGNPs), monocalcium phosphate monohydrate (MCPM, Himed, Old Bethpage, NY, USA), and andrographolide (Nanjing NutriHerb BioTech, Jiangsu, China). Five experimental formulations with varying concentrations of Sr-BGNPs/MCPM (Sr/Ca) and Andro were prepared (Table 1). The powder and liquid phases were mixed using a powder to liquid ratio of 4:1 (mass ratio). The mixed adhesives were then loaded in a composite syringe (MIXPAC 1 mL syringe, medmix Switzerland AG, Haag, Switzerland). Commercial resin composite orthodontic adhesive (Trans, Transbond XT, 3M-ESPE, Seefeld, Germany) was used as a control.

**Table 1:** Formulations of the experimental orthodontic adhesives.

Component/formulations		S10A10 (wt%)	S10A5 (wt%)	S5A10 (wt%)	S5A5 (wt%)	S0A0 (wt%)
Boroaluminosilicate glass (particle diameter ~ 7 µm)		40	42.5	42.5	45	50
Boroaluminosilicate glass (particle diameter ~ 0.7 µm)		40	42.5	42.5	45	50
Sr/CaP	Sr-BGNPs (diameter ~ 200 nm)	5	5	2.5	2.5	0
	MCPM (diameter ~ 10 µm)	5	5	2.5	2.5	0
Andro (particle diameter ~ 20–50 µm)		10	5	10	5	0

### Degree of monomer conversion

The uncured adhesives (n = 5) were placed in a metal circlip (1 mm in thickness and 10 mm in diameter) over the diamond of attenuated total reflection (ATR, iD7 ATR, Thermo Fisher Scientific, Waltham, MA, USA) of a Fourier transform infrared spectroscope (FTIR, Nicolet iS5, Thermo Fisher Scientific). The materials were then covered with an acetate sheet and light-activated for 20 s using an LED light-curing unit (irradiance of 1200 mW/cm^2^, SmartLite Focus Pen Style, DENTSPLY Sirona, York, PA, USA) at a distance of 1–2 mm from the top of the surface of each material. The FTIR spectra in the region of 700–4000 cm^−1^ were recorded from the bottom of each material. The degree of monomer conversion (DC) was then calculated using the following equation^12^.

1 $${\text{DC }} = { }\frac{{100\left( {\Delta {\text{A}}_{0} - \Delta {\text{A}}_{{\text{t}}} } \right)}}{{\Delta {\text{A}}_{0} }}$$

where $\Delta {\text{A}}_{0}$ and $\Delta {\text{A}}_{{\text{t}}}$ are the absorbance of the C–O peak (1320 cm^−1^)^28^ above the baseline at 1335 cm^−1^ before and after curing at time *t*, respectively.

### Biaxial flexural strength and modulus of elasticity

Disc specimens were prepared (n = 8). The composites were placed in a metal ring (10-mm in diameter and 1-mm in thickness). The materials were covered with an acetate sheet and glass slides on the top and bottom sides. The specimens were cured by an LED light-curing unit for 20 s on both sides. They were left for 24 h at 25 ± 1 °C. Then, the specimens were placed into 10 mL of deionized water at 37 °C. The biaxial flexural strength (BFS) test was performed using a ball-on-ring testing jig with a universal testing machine (AGSX, Shimadzu, Kyoto, Japan). The specimen was loaded with a 500 N load cell with a crosshead speed of 1 mm/min until the specimen failed. The BFS (Pa) was calculated using the following equation^29^.

2 $${\text{BFS }} = { }\frac{{\text{F}}}{{{\text{d}}^{2} }}\left\{ {\left( {1 + {\text{a}}} \right)\left[ {0.485{\text{ln}}\left( {\frac{{\text{r}}}{{\text{d}}}} \right) + 0.52} \right] + 0.48} \right\}$$

where F is the failure load (N), d is the thickness of the sample (m), r is the radius of circular support (mm), and a is Poisson’s ratio (0.3). Additionally, the biaxial flexural modulus (BFM, Pa) was calculated using the following equation.

3 $${\text{BFM }} = { }\left( {\frac{{{\Delta H}}}{{{\Delta W}_{{\text{c}}} }}} \right) \times \left( {\frac{{{\upbeta }_{{\text{c}}} {\text{d}}^{2} }}{{{\text{q}}^{3} }}} \right)$$

where $\frac{{{\Delta H}}}{{{\Delta W}_{{\text{c}}} }}$ is the rate of change of the load with regard to the central deflection or gradient of force versus the displacement curve (N/m), ${\upbeta }_{{\text{c}}}$ is the center deflection junction (0.5024), and q is the ratio of the support radius to the radius of the disc.

### Water sorption and solubility

Disc specimens (n = 6) were prepared and weighed using a four-figure balance. The test was performed according to BS EN ISO 4049^30^. The disc specimens were placed in a desiccator and kept in an incubator at the controlled temperature of 37 ± 1 °C for 22 h. Then, the desiccator was kept at room temperature (25 ± 1 °C) for ~ 2 h. The weight of each specimen was measured until a constant mass (m_1_) was obtained. The specimens were then placed in 10 mL of deionized water at 37 ± 1 °C for up to 4 weeks. Each specimen’s mass was then recorded (m_2_). Specimens were then reconditioned following the steps described above for m_1_ until a constant mass was obtained (m_3_). The water sorption (W_SP_, g/m^3^) and water solubility (W_SL_, g/m^3^) were calculated using the following equations.

4 $${\text{W}}_{{\text{SP }}} = { }\frac{{{\text{m}}_{2} - {\text{m}}_{3} }}{{\text{V}}}$$

5 $${\text{W}}_{{{\text{SL}}}} { } = { }\frac{{{\text{m}}_{1} - {\text{m}}_{3} }}{{\text{V}}}$$

where m_1_ is the conditioned mass of the specimen (g), m_2_ is the mass of the specimen after immersion in water for 4 weeks (g), m_3_ is the reconditioned mass of the specimen after immersion in water (g), and v is the volume of the specimen (m^3^).

### Enamel shear bond strength (SBS) and adhesive remnant index (ARI score)

The use of extracted teeth was approved by the Ethics Review Subcommittee Board for Human Research Involving Sciences, Thammasat University (No. 3, Faculty of Health Sciences and Science and Technology, Project No. 151/2563, approval date 11th November 2020). The thirty extracted premolars were collected at Thammasat University Hospital, Pathum Thani, Thailand. Consent from patients was waived by the Ethics Review Subcommittee Board for Human Research Involving Sciences, Thammasat University (No. 3, Faculty of Health Sciences and Science and Technology) because patient identification of the extracted teeth was not needed.

The extracted teeth were kept in 0.1% thymol solution at room temperature for less than 30 days prior to the test (n = 5). The root was cut at 2 mm under the cervical line. The buccal surface was conditioned with 37% phosphoric acid (Transbond™ XT etching gel; 3 M-ESPE) and rinsed for 15 s followed by gentle air-drying. The etched surface received a primer application (Transbond™ XT Light Cure Orthodontic Primer, 3 M-ESPE) for 10 s and was then air-dried. The experimental adhesives were then applied on the primed surface, followed by the placement premolar brackets (GEMINI MBT 0.022 Twin, 3 M-ESPE). The excess adhesive was removed. Then, the specimen was light-cured using an LED light-curing unit for 10 s on the mesial and distal sides.

Specimens were embedded in a self-cured acrylic resin in a polyvinyl chloride (PVC) tube and immersed in artificial saliva^31^ for 24 h. Then, the specimens were subjected to thermocycling (5 and 55 °C) with an immersion time and dwell time of 30 s and 10 s for 500 cycles according to PD ISO/TS 11405:2015 ^32^. Then, the specimens were placed in a shear-bond-strength testing jig under a mechanical testing frame. A knife-edge chisel was applied at the interface between the tooth and the bracket. The specimens were loaded with a 500 N load cell at a crosshead speed of 1 mm/min. The maximal load (F, Newton) before the debonding of the bracket was recorded. The shear bond strength of materials to enamel (SBS, Pa) was then calculated using the following equation^12^.

6 $${\text{SBS }} = { }\frac{{\text{F}}}{{\text{A}}}$$

where A is the area of the bonding interface (m^2^). An adhesive remnant index was determined by observing the residual adhesive on the brackets under a stereomicroscope (10 × magnification). The ARI index was classified into four categories as follows^33,34^.

- Score 0: no adhesive remained on the enamel.
- Score 1: less than 50% of the adhesive remained on the enamel surface.
- Score 2: more than 50% of the adhesive remained on the enamel surface.
- Score 3: all adhesive remained on the enamel surface.

### Calcium phosphate precipitation

The test was performed according to the protocol used in the previous study^12^. The specimen (n = 1) was prepared according to the SBS test. The specimens were placed in 10 mL of artificial saliva at 37 °C for 24 h. Then, the brackets were de-bonded from the tooth. The surface of detached bracket was then coated with Au using a sputter-coating machine with a current of 23 mA for 45 s. The precipitation of calcium phosphate crystals on the surface was assessed under a scanning electron microscope (SEM, JSM, 7800F, JEOL Ltd., Tokyo, Japan). Then, the elemental composition of the precipitation was analyzed using an energy dispersive X-ray analysis (EDX, X-sight 6650 detector, Oxford Instruments, Abingdon, UK) with a beam voltage set at 10 kV.

### Ion release

Disc specimens (n = 3) were prepared and placed in 10 mL of deionized water. The tubes were incubated at 37 °C for 4 weeks. The storage solution was then collected to analyze the concentrations of Ca, P, and Sr ions. The extract was mixed with 3 vol% nitric acid. The concentrations of ions were assessed using inductively coupled plasma–atomic emission spectroscopy (ICP–OES, Optima 8300, PerkinElmer, Waltham, MA, USA).

### Influence on the growth of S. mutans

*Streptococcus mutans* (ATCC 25175) was inoculated in Mueller Hinton (MH) broth (BD Difco™ Mueller Hinton Broth, Thermo Fisher Scientific Inc., Göteborg, Sweden) using a 1:2 volume ratio of inoculum to broth^12^. They were incubated for 24 h at a controlled temperature of 37 °C and enriched with 5% CO_2_. The suspension of *S. mutans* was then adjusted until a bacterial concentration of 2.5 × 10^5^ cells/mL was obtained using a spectrophotometer at an optical density (OD) of 600 nm.

Disc specimens (1 mm in thickness and 10 mm in diameter) were prepared and sterilized through UV irradiation for 30 min on each surface (n = 3)^35^. The discs were then placed in tubes containing mixtures of 2 mL of Mueller Hinton Broth and 1 mL of the suspension of *S. mutans*. A tube without a disc specimen was used as the blank control. The tubes were incubated at a controlled temperature of 37 °C in air enriched with 5% CO_2_ for 48 h. Then, the discs were removed. The suspensions were vortexed for 30 s, followed by serial dilution until bacterial concentrations of 1 × 10^–6^ CFU/mL were obtained. The suspensions (200 µL) were then plated on Mitis Salivarius agar and incubated at 37 °C under a 5% CO_2_ atmosphere for 48 h. Colony-forming units (log CFU/mL) were then counted using a microscope and image analysis.

### Statistical analysis

Numerical data are reported as the mean and SD. The results were analyzed using Prism version 9.3 for macOS (GraphPad Software, San Diego, CA, USA). The normality of the data distribution was evaluated using the Shapiro–Wilk test. BFS and ion release results were compared using one-way ANOVA followed by Tukey’s multiple comparisons test. DC, BFM, W_sp_, W_SL_, SBS, and antibacterial tests were compared using the Kruskal–Wallis test followed by the Dunn test. A chi-squared test was used to evaluate the ARI scores among the adhesive subgroups. Statistical significance was set at *p* = 0.05. The sample size used in each test was calculated by G*Power 3.1 software (University of Dusseldorf, Dusseldorf, Germany) using the results in published studies and a pilot study^10,12,29^. The result indicated that the sample size in each test gave power > 0.95 at alpha = 0.05. Additionally, factorial analysis was performed to assess the effects of increasing the concentrations of Sr/CaP (5 to 10 wt%) and Andro (5 to 10 wt%) on the tested properties ^10^.

## Results

### Characterization of Sr-bioactive glass nanoparticles (SrBGNPs)

**Figure 1:**
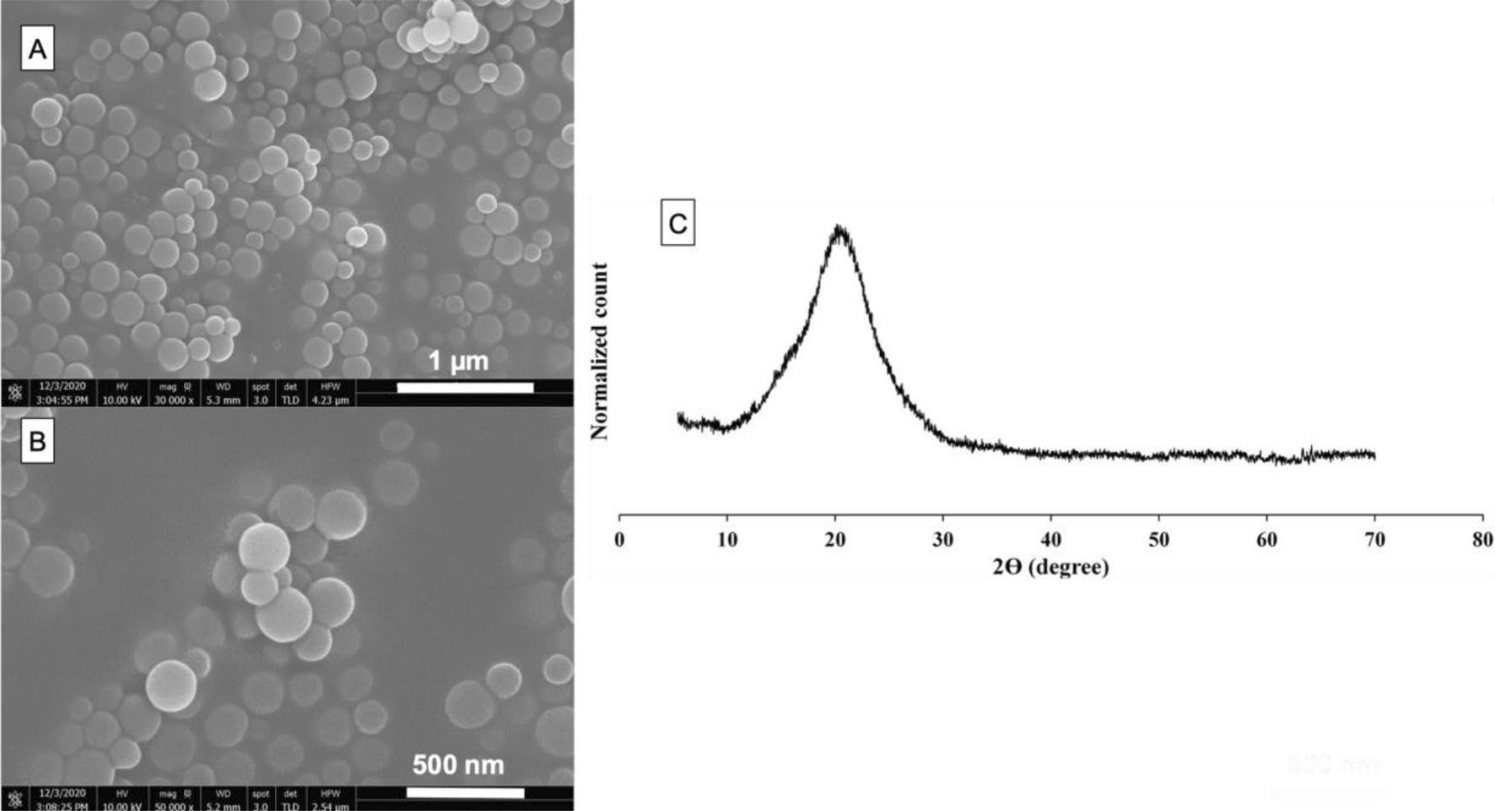
(**A**,**B**) SEM images and (**C**) XRD pattern of the Sr-bioactive glass nanoparticles (Sr-BGNPs).

Monodisperse spherical Sr-BGNPs were successfully synthesized in the 170 ± 30-nm-diameter range through a sol–gel process (Fig. 1A,B). The elemental composition of the Sr-BGNPs was 81.0 mol% SiO_2_, 14.2 mol% CaO, and 4.8 mol% SrO. The XRD pattern of the Sr-BGNPs showed a broad halo of particles calcined at 680 °C, indicating that calcium oxide (CaO) and strontium oxide (SrO) were successfully incorporated into the amorphous structure (Fig. 1C).

### Degree of monomer conversion (DC)

**Figure 2:**
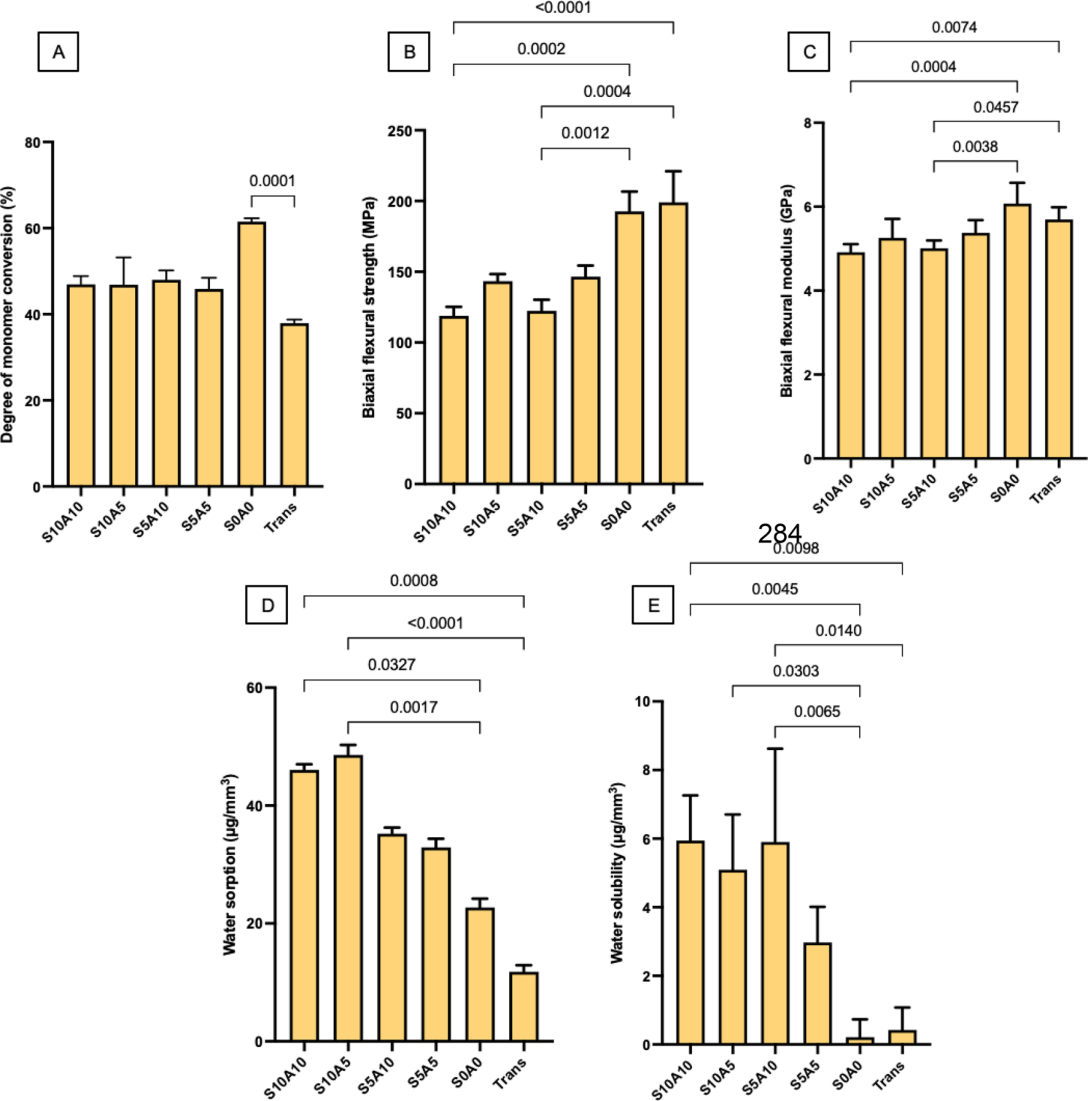
(**A**) Degree of monomer conversion after light-curing for 20 s (mean ± SD, n = 5). (**B**) Biaxial flexural strength (BFS) and (**C**) biaxial flexural modulus (BFM) after immersion in deionized water for 24 h (mean ± SD, n = 8). (**D**) Water sorption and (**E**) water solubility after immersion in deionized water for 4 weeks (mean ± SD, n = 6). The lines indicate *p* < 0.05.

The highest and lowest DCs were obtained from specimens S0A0 (62 ± 1%) and Trans (38 ± 1%), respectively (Fig. 2A). The DCs of specimens S10A10 (47 ± 2%), S10A5 (47 ± 6%), S5A10 (48 ± 2%), and S5A5 (46 ± 2%) were comparable (*p* > 0.05). The conversion of S0A0 was significantly higher than that of Trans (*p* < 0.01). Factorial analysis indicated that increases in the Sr/CaP and Andro concentrations from 5 to 10 wt% showed negligible effects on the DCs of the materials.

### Biaxial flexural strength (BFS) and modulus of elasticity (BFM)

The highest and lowest BFSs were obtained from Trans (199 ± 22 MPa) and S10A10 (119 ± 6 MPa), respectively (Fig. 2B). The BFSs of Trans and S0A0 (193 ± 10 MPa) were significantly higher than those of S10A10 and S5A5 (147 ± 22 MPa) (*p* < 0.05). The BFSs of S0A0, S10A5 (143 ± 5 MPa), S5A10 (122 ± 8 MPa), and S5A5 (147 ± 8 MPa) were comparable (*p* > 0.05). Factorial analysis indicated that an increase in Andro from 5 wt% to 10 wt% reduced the BFS by 17 ± 4%. However, an increase in the level of Sr/CaP showed negligible effects on the BFSs of the experimental materials.

The highest and lowest BFMs were obtained from S0A0 (6.1 ± 0.5 GPa) and S10A10 (4.9 ± 0.2 GPa) (Fig. 2C). The BFMs of S10A5, S5A10, S5A5, and Trans were 5.3 ± 0.5 GPa, 5.0 ± 0.2 GPa, 5.4 ± 0.3 GPa, 5.7 ± 0.3 GPa, respectively. The BFM of Trans was comparable to those of S5A10 (*p* = 0.461), S5A5 (*p* = 0.541), and S0A0 (*p* = 0.716). The BFMs of S10A10 and S5A10 were significantly lower than those of S0A0 (*p* < 0.05) and Trans (*p* < 0.01). Factorial analysis showed that an increase in Andro from 5 wt% to 10 wt% reduced the BFM by 10 ± 8%. However, an increase in Sr/CaP showed negligible effects.

### Water sorption and solubility

The highest and lowest water sorption values were obtained from S10A5 (48.6 ± 1.5 μg/mm^3^) and Trans (12.0 ± 1.0 μg/mm^3^), respectively (Fig. 2D). The water sorption values of S10A10, S5A10, S5A5 and S0A0 were 46.0 ± 0.9 μg/mm^3^, 35.2 ± 1.0 μg/mm^3^, 32.9 ± 1.4 μg/mm^3^, and 22.7 ± 1.4 μg/mm^3^, respectively. The water sorption values of S10A10 and S10A5 were significantly higher than those of S0A0 (*p* < 0.05) and Trans (*p* < 0.01). Factorial analysis indicated that an increase in Sr/CaP from 5 to 10 wt% enhanced water sorption by 39 ± 4%, while the effect of Andro was negligible.

The highest and lowest water solubilities were obtained from S10A10 (5.9 ± 1.3 μg/mm^3^) and S0A0 (0.2 ± 1.2 μg/mm^3^), respectively (Fig. 2E). The water solubilities of F2, F3, F4 and Trans were 5.1 ± 1.6 μg/mm^3^, 5.9 ± 1.9 μg/mm^3^, 3.0 ± 1.0 μg/mm^3^, and 0.4 ± 1.0 μg/mm^3^, respectively. F1, F2 and F3 showed significantly higher water solubilities than F5 (*p* < 0.05). F1 and F3 also showed significantly higher water solubilities than Trans (*p* < 0.05). The factorial analysis indicated that an increase in Andro from 5 to 10 wt% increased water solubility by 55.97 ± 55.91%, while the effect of Sr/CaP was minimal.

### Enamel shear bond strength (SBS) and adhesive remnant index (ARI score)

**Figure 3:**
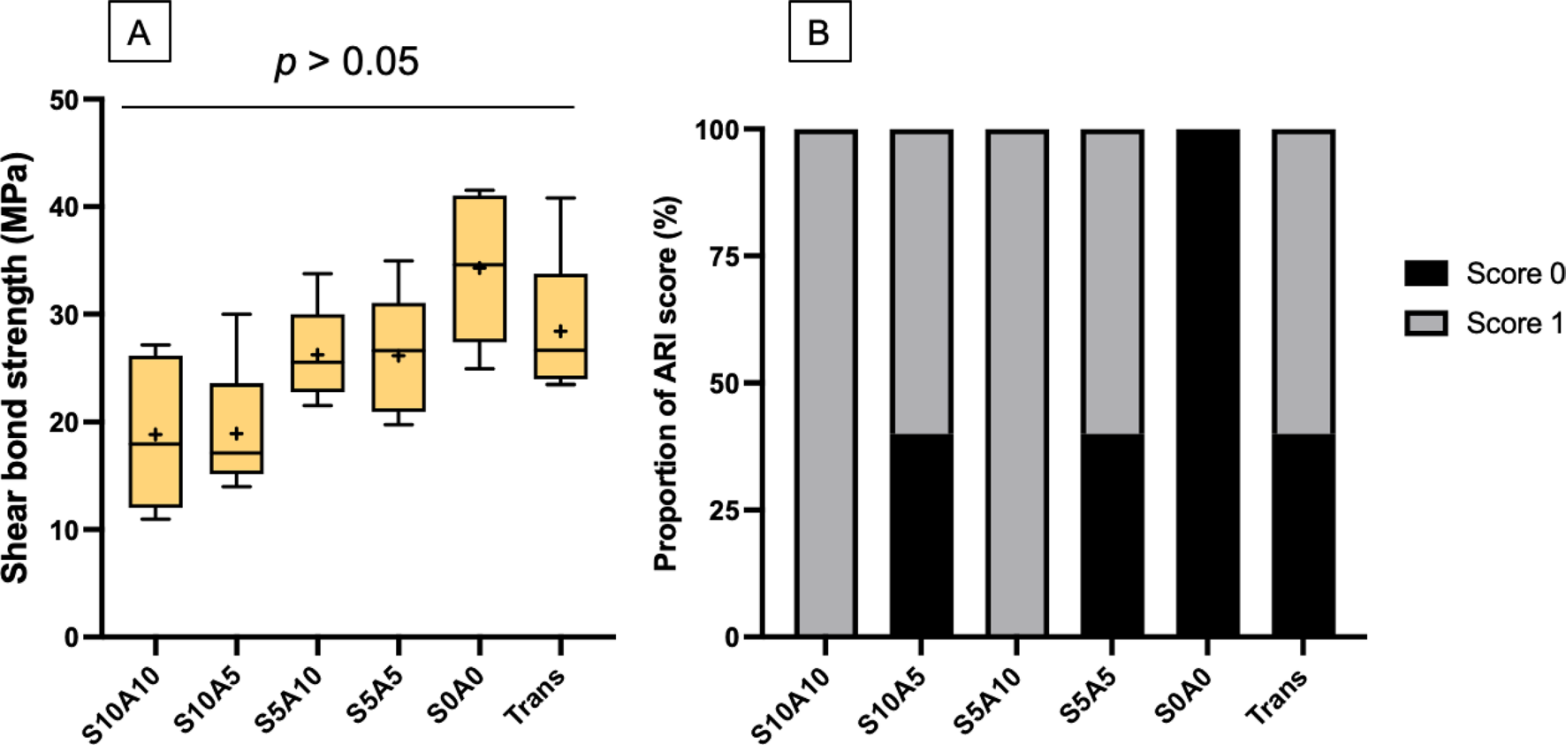
(**A**) Enamel shear bond strengths of materials. The boxes represent the first quartile (Q1) to the third quartile (Q3), the horizontal lines in the box represent the median, the whiskers represent the maximum and minimum values, and “ + ” represents the mean value (n = 5). (**B**) Proportions of the adhesive remnant indices (ARI scores) of the specimens in each group. The ARI scores of 2 and 3 were not detected from the specimens.

The highest and lowest SBSs (median, min–max) were obtained from S0A0 (34.6, 24.9–41.5 MPa) and S10A10 (18.0, 11.0–27.1 MPa) (Fig. 3A). The SBS of Trans (26.6, 23.5–40.8 MPa) was similar to those of S10A10, S10A5 (17.12, 14.0–30.0 MPa), S5A10 (25.5, 21.5–33.8 MPa), S5A5 (26.6, 19.8–35.0 MPa), and S0A0 (*p* > 0.05). The factorial analysis demonstrated that an increase in Sr/CaP from 5 to 10 wt% reduced SBS by 28 ± 22%. The effect of Andro was negligible. The most common ARI scores observed from all materials were scores of 0 and 1 (Fig. 3B). The ARI scores of 2 and 3 were not observed from the specimens. The distribution of the score among each group was not similar (*p* < 0.05).

### Calcium phosphate precipitation

**Figure 4:**
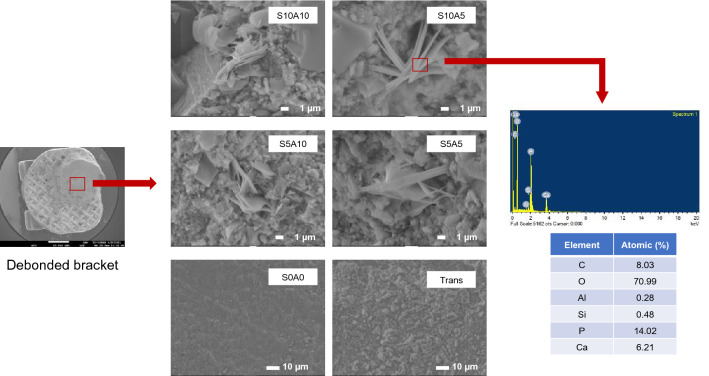
Surfaces of the adhesives of the representative specimens after debonding from enamel. Precipitates (arrows) were detected in all groups except for S0A0 and Trans. An example of the EDX results from the precipitation showed that the main elements contained in the precipitate crystals are Ca and P.

The precipitation of calcium phosphate crystals was detected on the debonded surfaces of the brackets of all groups except for S0A0 and Trans. The EDX results demonstrated that the precipitate contained Ca and P (Fig. 4).

### Ion release

**Figure 5:**
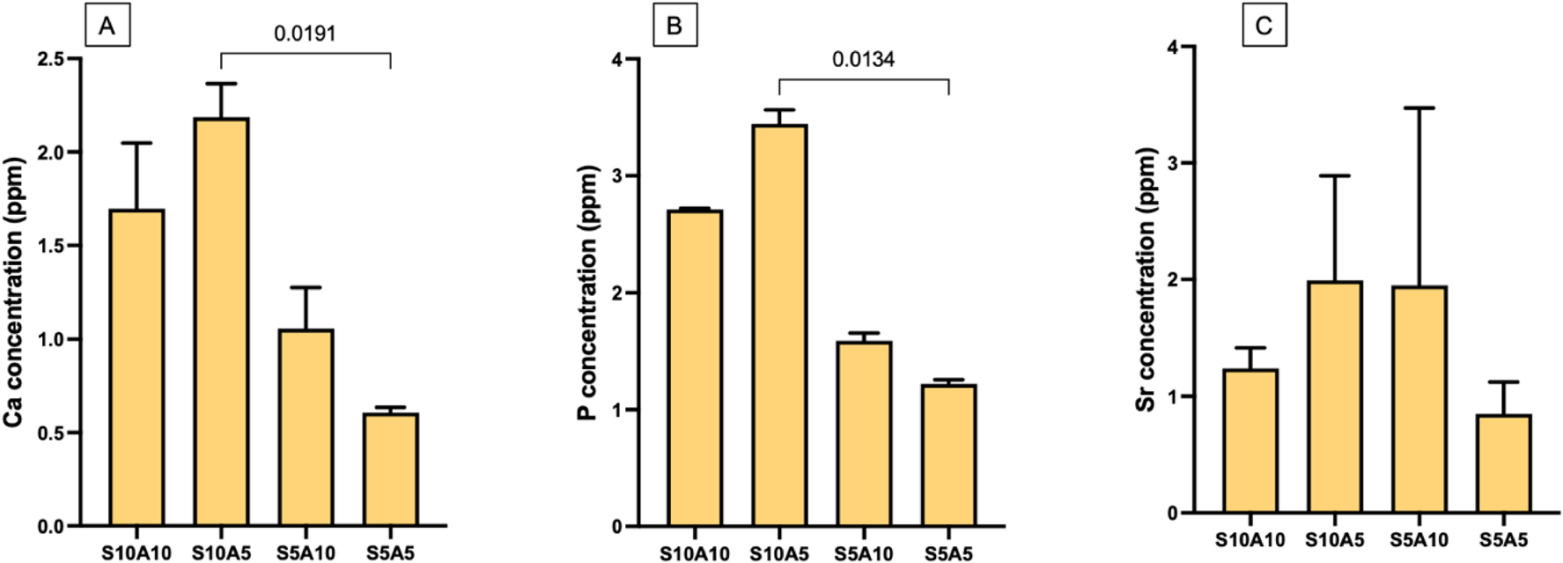
Concentrations of (**A**) calcium, (**B**) phosphorus, and (**C**) strontium ions contained in the storage solution at 4 weeks (mean ± SD, n = 3). The amounts of released ions from S0A0 and Trans were under the detection limit. Lines indicate *p* < 0.05.

Ca, P, and Sr ions were not detected from S0A0 and Trans. The highest and lowest concentrations of Ca ions were detected from S10A5 (1.70 ± 0.29 ppm) and S5A5 (0.61 ± 0.02 ppm) (Fig. 5A). For P, the highest and lowest concentrations were detected from S10A5 (3.44 ± 0.10 ppm) and S5A5 (1.22 ± 0.03 ppm) (Fig. 5B). Likewise, the highest and lowest Sr concentrations were detected from S10A5 (1.99 ± 0.73 ppm) and S5A5 (0.85 ± 0.22 ppm) (Fig. 5C). The factorial analysis demonstrated that an increase in Sr/CaP from 5 to 10 wt% increased the amounts of released Ca and P by 141 ± 19% and 119 ± 4%, respectively. The effects of increasing the concentrations of Sr/CaP and Andro on the release of Sr were negligible.

### Influence on the growth of S. mutans

**Figure 6:**
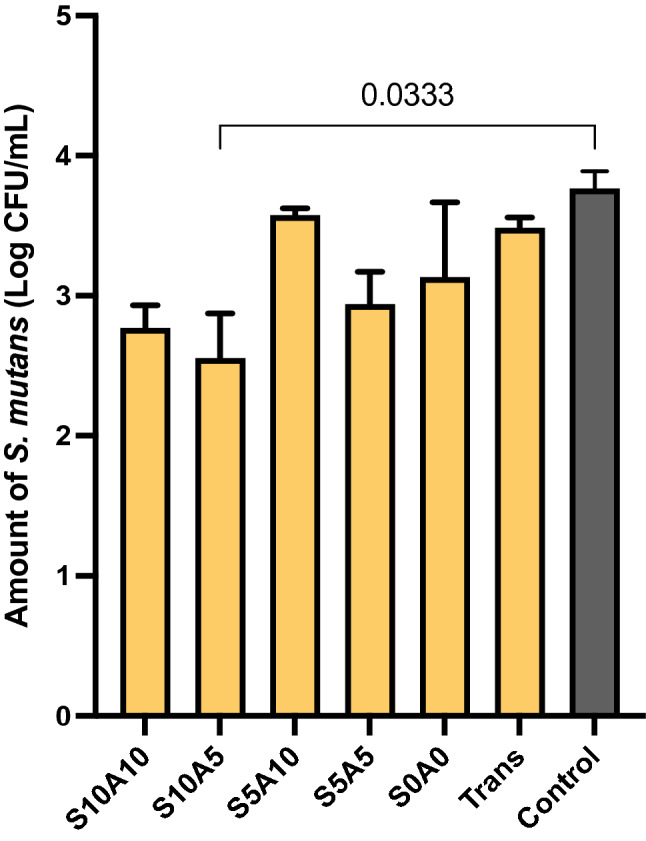
Amounts of *S. mutans* in all materials compared to the blank control (no material) (mean ± SD, n = 3). The amounts of released ions from S0A0 and Trans were under the detection limit. Lines indicate *p* < 0.05.

The highest and lowest amounts of *S. mutans* were detected from the blank control (3.8 ± 0.1 Log CFU/mL) and S10A5 (2.6 ± 0.3 Log CFU/mL) (Fig. 6). The value of S10A5 was significantly lower than that of the blank control (*p* < 0.01). The amount of *S. mutans* in S10A5 was comparable to those in S10A10 (2.8 ± 0.1 Log CFU/mL), S5A10 (3.6 ± 0.1 Log CFU/mL), S5A5 2.9 ± 0.2 Log CFU/mL, S0A0 (3.1 ± 0.5 Log CFU/mL), and Trans (3.5 ± 0.1 Log CFU/mL) (*p* > 0.05). Factorial analysis showed that an increase in Sr/CaP from 5 to 10 wt% reduced the Log CFU/mL of *S. mutans* by 18 ± 3%. An increase in Andro from 5 to 10 wt% showed no reduction in the amount of *S. mutans*.

## Discussion

The limitation of the commonly used resin-based orthodontic adhesive is the lack of ion release and antibacterial actions resulting in the formation of white spot (early caries) lesions around brackets, which is a common esthetic complication during orthodontic treatment. The aim of the current study was to prepare experimental orthodontic adhesives containing Sr/CaP and Andro for promoting ion release and antibacterial action, which could potentially help reduce the risk of white spot lesions. The effects of Sr/CaP and Andro on the physical/mechanical and antibacterial actions of the adhesives were then determined. The research hypothesis was rejected because increases in the concentrations of additives significantly affected the water sorption/solubility, biaxial flexural strength and modulus, shear bond strength, mineral precipitation, ion release, and growth of *S. mutans* of the experimental adhesives.

High degrees of monomer conversion of orthodontic adhesives are required to reduce the risk of toxic monomer leaching and provide sufficient adhesive mechanical strength^36^. The release of unbound monomers due to the suboptimal polymerization of orthodontic adhesives could potentially enhance the cariogenicity of dental biofilms^5^. The additives reduced the DC of the experimental orthodontic adhesives, which could be due to an increase in refractive index mismatch^10^. This may increase light scattering and reduce light penetration and polymerization at the inner surface. The higher DCs of the experimental materials compared to that of Trans could be due to the differences in the primary methacrylate monomer. Bisphenol A-glycidyl methacrylate (Bis-GMA) is the primary base monomer of Trans. The glass transition temperature (*T*_*g*_) of UDMA (− 35.3 °C), which was the primary base monomer in the experimental material, is lower than that of Bis-GMA (− 7.7 °C). The polymer containing monomers with a low glass transition temperature (*T*_*g*_) usually attains a higher level of monomer conversion compared to polymers consisting of high-*T*_*g*_ monomers^37,38^. A higher monomer conversion was expected to reduce the risk of monomer release^39^. However, elution studies and toxicity tests should be included in future work.

Resin-based materials can absorb water from the environment, leading to hygroscopic expansion, hydrolytic degradation, and the release of the active components. Excessive water sorption may cause water plasticization, which could subsequently reduce the strength of the materials^40,41^. Additionally, water sorption may lead to the volume expansion of resin-based material, which could help compensate for the polymerization shrinkage stress that occurred within the material^42^. The W_sp_ values of S10A10 and S10A5 were higher than the level required by BS ISO 4049 (< 40 µg/mm^3^). This could be due to the hydrophilicities of MCPM and the Sr-BGNPs. However, the W_sp_ values were much lower than those of previously developed Ca/P-releasing orthodontic adhesives containing 10 wt% MCPM^12^. This could be because the concentration of MCPM (2.5–5 wt%) used in the current study was lower than that used in the previous study. The water sorption values of all experimental adhesives were within the range of that required by BS ISO 4049 for luting-type dental composites (< 7.5 µg/mm^3^). Previous studies reported that the addition of MCPM promoted ion release and the formation of calcium phosphate fillers, such as dicalcium phosphate dihydrate (DCPD), the structure of which contains water^43,44^. The formation of dicalcium phosphate in the materials may help decrease mass loss and water dissolution. Other possible explanations include the slow degradation of strontium-bioactive glass nanoparticles and the low water solubility of andrographolide.

Currently, there are no minimum requirements specified by the ISO for the enamel shear bond strengths of orthodontic adhesives. A high SBS is essential for ensuring that the active force from the archwire can be transferred to the anchored tooth without dislodgement. Although the SBSs of the experimental adhesives were lower than that of Trans, the values were still higher than the minimum clinically acceptable SBS (5.9–7.8 MPa)^45^. It is speculated that the addition of nonsilanized fillers may detrimentally reduce the SBSs of the experimental adhesives by the accelerated degradation of the matrix-filler interface during thermocycling. The SBSs of the experimental adhesives obtained from the experimental materials were still within the range or higher than that reported in published studies (6–32 MPa)^46,47^. This could be due to the use of a minimum number of aging cycles (500 cycles) according to the ISO (British Standard PD ISO/TS 11405:2015 Dentistry-Testing of adhesion to tooth structure) in the current study^32^. A larger number of aging cycles, i.e., 5,000–30,000 cycles, should be used in future studies to ensure the long-term bonding performance of the materials ^48^.

Although the observed SBS values among the experimental adhesives were similar, the factorial analysis indicated that the incorporation of Sr/CaP reduced the SBSs of the materials by ~ 28%. The reduction in SBS upon the addition of reactive fillers could be due to the lack of silanization of the additives or the hydrophilicity of the Sr/CaP fillers^49,50^. It is speculated that the main effect could be primarily from MCPM due to its high water solubility (~ 18 g/L at 25 °C)^11,44,50,51^. Hence, future work may need to assess the formulation with low-level MCPM (1–2 wt%) or no MCPM (0 wt%). This could additionally help reduce the excessive water sorption/solubility of the experimental materials.

The adhesive remnant index (ARI, 0 to 4) is one of the most commonly used methods for assessing the characteristics of the adhesion between the adhesive and enamel. A high ARI score may indicate excessively strong adhesion between the adhesive and enamel surface, which could lead to enamel fracture at the interface. Hence, failure within the adhesive layer (low ARI score) during the detachment of brackets is preferred to reduce the risk of the breakdown of the enamel surface. The ARI scores of the materials in the current studies were mostly 0 or 1, which was similar to that of the published studies^52,53^. This may be desirable to help preserve the enamel surface during the debonding of brackets.

The addition of Sr/CaP fillers promoted the release of Ca, P, and Sr ions. These ions were expected to promote suitable conditions for the precipitation of biological hydroxyapatite to enhance remineralization. The experimental adhesives containing high Sr/CaP concentrations (S10A10, S10A5) showed greater amounts of released Ca and P ions compared to formulations with lower Sr/CaP concentrations. A possible explanation for this could be that the release of Ca and P ions was primarily due to the dissolution of MCPM. The differences in Sr^2+^-release among groups were not clearly observed, which may be due to the lower solubility of the Sr-BGNPs. All experimental adhesives containing Sr/CaP promoted the precipitation of calcium phosphate at the interface. The EDX results showed that the Ca/P atomic ratio of the precipitate (0.5) on the debonded brackets was much lower than that of hydroxyapatite (1.67). This may suggest that the precipitate could be representative of the early stage of calcium phosphate crystal formation because the immersion time of the specimen was only 24 h. The Ca/P ratio may be increased with an increase in the immersion time^54^. A previous study demonstrated that the precipitation of hydroxyapatite could be detected on an orthodontic adhesive containing bioactive glass after immersion in artificial saliva for 6 months^55^. A longer immersion time to confirm the ability of the materials to promote apatite formation may be employed in future studies.

The increase in the level of Sr/CaP from 5 to 10 wt% (S10A10, S10A5) enhanced the growth inhibition of the planktonic *S. mutans* exhibited by the experimental orthodontic adhesives. It was speculated that the bacteriostatic action observed with the experimental orthodontic adhesives could be primarily due to the Sr-BGNPs since the potential benefits of Sr^2+^-release include both remineralizing and antibacterial actions^18^. This result was in accordance with those of previous studies that demonstrated the antibacterial actions of Sr-containing bioactive glasses^56,57^. It was speculated that the growth inhibition of *S. mutans* could be due to the release of Sr-BGNPs from the experimental adhesives due to the lack of silanization. The large surface-to-volume ratio and high charge density of the nanoparticles may facilitate interactions with negatively charged bacterial cell membranes, thereby increasing the antimicrobial activity of the nanoparticles even at low concentrations^58^. The current study, however, failed to demonstrate the growth inhibition of planktonic *S. mutans* by Andro. A possible explanation for this could be due to the low solubility of Andro^59^, which may limit its release from the adhesives. Additionally, it was proposed that the antibacterial action of Andro mainly involved the inhibition of the biofilm through Andro acting as a quorum-sensing inhibitor that interfered with the biofilm-forming process^60^. Furthermore, it should be mentioned that the bacterial growth inhibition may be due to the unreacted monomers released from the materials^61^. Hence, the monomer elution study should be examined in future work.

It should be mentioned that the current study was an in vitro study, so the clinical relevance should be carefully interpreted. The results from this preliminary study indicate that the formulation with a high level of Sr/CaP (S10A10 and S10A5) generally exhibited desirable and acceptable results except for water sorption/solubility. Further characterization and modification of the formulations may be needed in future works.

## Conclusion

Experimental orthodontic adhesives containing MCPM/Sr-BGNPs and Andro were developed for promoting ion release and inhibiting cariogenic bacteria. The additives reduced the physical and mechanical properties of the materials, but the corresponding values were still within the acceptable range. The addition of Sr/CaP promoted calcium phosphate precipitation and the inhibition of planktonic *S. mutans*. The increase in Andro reduced the strengths of the materials and failed to demonstrate antibacterial actions. These promising properties of the experimental orthodontic adhesives were expected to help reduce the risk of developing white spot lesions around excessive amounts of adhesive for high caries-risk patients.

## Data Availability

The datasets generated and/or analyzed during the current study are available from the corresponding author upon reasonable request.
